# Interactions and CCAAT-Binding of *Arabidopsis thaliana* NF-Y Subunits

**DOI:** 10.1371/journal.pone.0042902

**Published:** 2012-08-17

**Authors:** Valentina Calvenzani, Barbara Testoni, Giuliana Gusmaroli, Mariangela Lorenzo, Nerina Gnesutta, Katia Petroni, Roberto Mantovani, Chiara Tonelli

**Affiliations:** Dipartimento di Bioscienze, Università degli Studi di Milano, Milano, Italy; Université Paris-Diderot, France

## Abstract

**Background:**

NF-Y is a transcription factor that recognizes with high specificity and affinity the widespread CCAAT box promoter element. It is formed by three subunits: NF-YA and the NF-YB/NF-YC- heterodimer containing histone fold domains (HFDs). We previously identified a large *NF-Y* gene family in *Arabidopsis thaliana*, composed of 29 members, and characterized their expression patterns in various plant tissues.

**Methods:**

We used yeast Two-hybrids assays (Y2H), pull-down and Electrophoretic Mobility Shift Assay (EMSA) *in vitro* experiments with recombinant proteins to dissect AtNF-YB/AtNF-YC interactions and DNA-binding with different AtNF-YAs.

**Results:**

Consistent with robust conservation within HFDs, we show that heterodimerization is possible among all histone-like subunits, including the divergent and related LEC1/AtNF-YB9 and L1L/AtNF-YB6 required for *embryo* development. DNA-binding to a consensus CCAAT box was investigated with specific AtNF-YB/AtNF-YC combinations and observed with some, but not all AtNF-YA subunits.

**Conclusions:**

Our results highlight (i) the conserved heterodimerization capacity of AtNF-Y histone-like subunits, and (ii) the different affinities of AtNF-YAs for the CCAAT sequence. Because of the general expansion of NF-Y genes in plants, these results most likely apply to other species.

## Introduction

The CCAAT box is one of the most ubiquitous promoter elements, being present in many, if not most of eukaryotic promoters [1]. Typically, it is found between −60 and −100 base-pairs from the transcriptional start site. The functional importance of the evolutionarily conserved consensus pentanucleotide has been widely established in several experimental systems. Twenty years of biochemical and genetic analyses have clarified that NF-Y [HAP2/3/5 in yeast] is a trimeric protein complex composed of NF-YA [HAP2], NF-YB [HAP3] and NF-YC [HAP5]. All subunits are required for DNA-binding and conserved throughout evolution [2]. NF-YB/NF-YC belong to the class of Histone Fold Domain [HFD] proteins, forming a tight dimer, structurally similar to H2A/H2B, with DNA-binding interaction modules [3]. Heterodimerization results in the formation of a surface for NF-YA association, allowing the resulting trimer to bind DNA with high specificity and affinity. The *fungi* HAP complex activates transcription through an additional subunit, HAP4, containing an acidic activation domain [4], [5], unlike the mammalian NF-YA and NF-YC subunits which display large domains rich in Glutamines with transcriptional activation potential [6], [7]. In plants, NF-Y also consists of three subunits and we and others have identified and classified them in *Arabidopsis* [8]–[10], and other species [11]–[15]. In general, plants have large families of genes, differentially expressed in various tissues: typically, 4–6 members are abundant and ubiquitous, while the others are restricted to certain tissues or developmental stages.

Genetic experiments were initially described for LEAFY COTYLEDON 1 (LEC1, AtNF-YB9) which has a role in *embryo* maturation and specification of cotyledon identity, with a unique pattern of expression confined to *embryos* ([16]–[18], reviewed in [19]). A LEC1 related member, L1L/AtNF-YB6, was shown to be able to partially complement the *lec1* defect [20], and chimeric constructs demonstrated that the HFD domain is necessary and sufficient for LEC1 function in *embryos* [17]. The LEC1 homologues have similar roles in carrot [21], [22] and *Theobroma cacao* [23]. Genetic analysis of AtNF-YA5 mutants indicate that it is involved in both ABA and blue light responses, together with LEC1 [24], and in drought resistance [25], similarly to AtNF-YB1 and YB2 and maize ZmNF-YB2 [26], [27]. AtNF-YB2 and AtNF-YB3 are important for flowering [28]–[31], and MtHAP2-1 regulates symbiotic nodules in *Medicago truncatula* [32].

The growing wealth of genetic data is poorly matched by biochemical advancements. The presence of 29 *bona fide* NF-Y genes in the *Arabidopsis* genome could potentially result in the formation of >900 alternative heterotrimeric combinations with different DNA-binding capabilities: the most obvious questions are whether there is specificity in interactions and whether all combinations are capable to bind to the CCAAT box. DNA-binding has been scored with carrot LEC1, one cNF-YB and two cNF-YCs [33], with OsHAP3A (NF-YB), six OsHAP5s (NF-YC) and one OsHAP2 [13], and AtNF-YB2 and AtNF-YB3 coupled to yeast HAP2 and HAP3 subunits [30]. A recent systematic study conducted on *Arabidopsis* NF-Y subunits using Y2H assays reached the following conclusions [34]: (i) the HFD subunits do not homodimerize, (ii) they heterodimerize among them, with a notable degree of specificity, and (iii) AtNF-YAs can only bind to HFD dimers, and not to single subunits. The last point was expected, given the wealth of previous biochemical and genetic work. To clarify the stunning complexity of this system, we undertook Y2H assays, *in vitro* pull-down and Electrophoresis Mobility Shift Assay (EMSAs), reporting the interaction map and DNA-binding activity of 24 members of the *Arabidopsis* NF-Y gene family.

## Results

### Yeast Two-Hybrids assays

**Figure 1:**
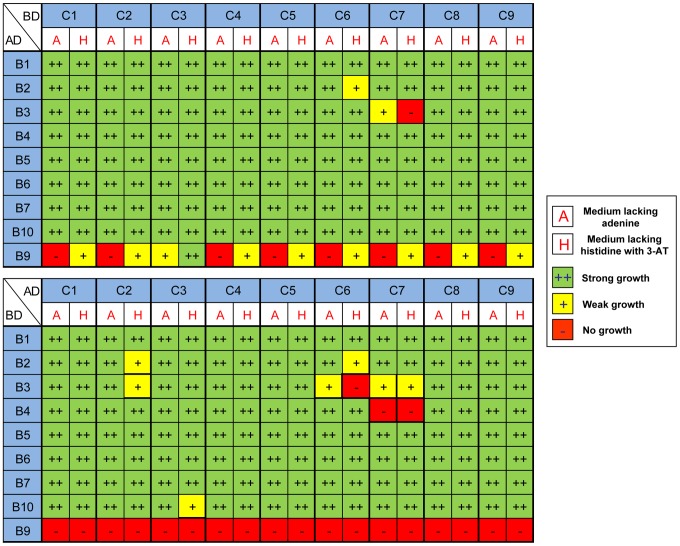
AtNF-YB-AtNF-YC interactions by colony yeast two hybrids assays. **A**.The indicated AtNF-YCs were fused to the Activation Domain (AD) and tested with AtNF-YB fused to the DNA-binding domain of GAL4. **B**. Same as A, except that the reverse experiment was tested, namely the AtNF-YBs fused to the Activation Domain were matched to the AtNF-YCs fused to the DNA Binding Domain. ++ refers to robust growth on the selective medium, + weak growth, and − no growth.

**Figure 2:**
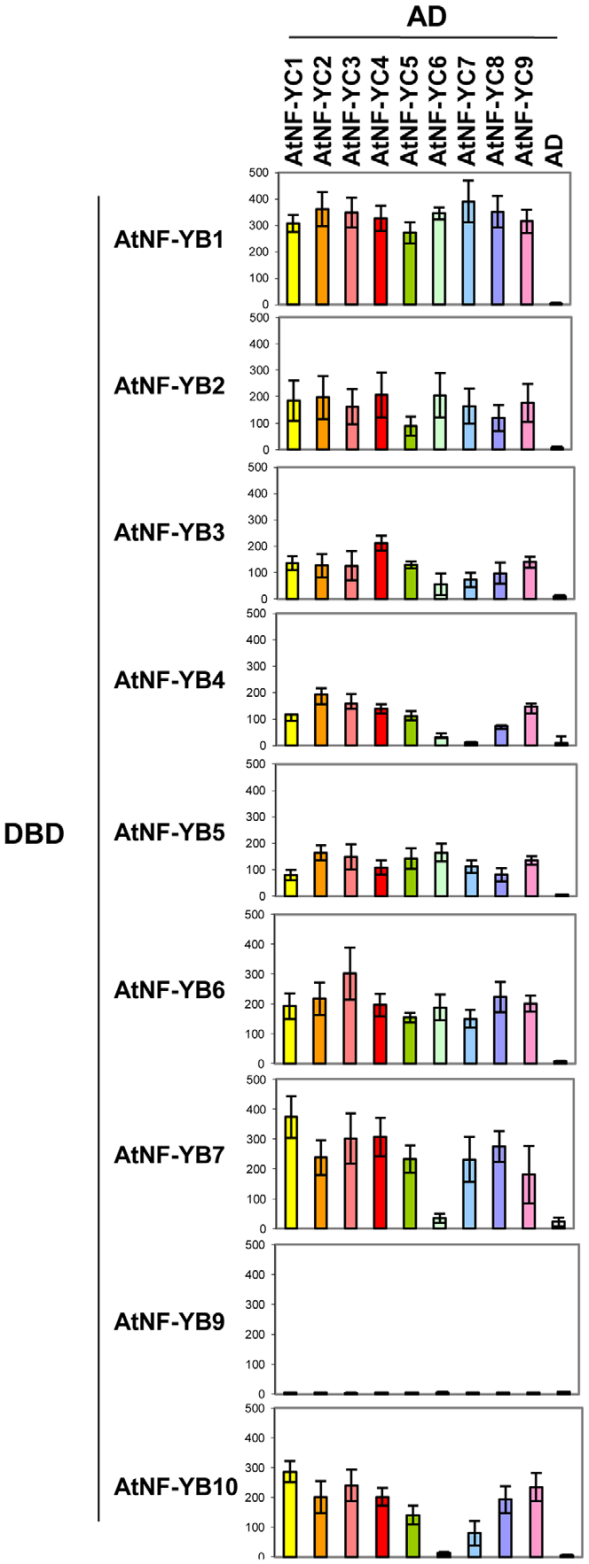
AtNF-YB-AtNF-YC interactions by liquid yeast two hybrids assays. Yeast two hybrids assays in liquid cultures using the AtNF-YB (DBD) and AtNF-YC (AD) configuration are depicted. β-Galactosidase Units were measured as detailed in Materials and Methods. The experiments were repeated three times and the standard deviations are indicated.

Since NF-YB and NF-YC are known to form a tight heterodimer, whose interaction generates an optimal surface for NF-YA association, we used Y2H assays to systematically dissect the ability of each member of the AtNF-YB and AtNF-YC family to interact with each other. The bait and prey vectors contained the GAL4 DNA-binding domain (DBD) and GAL4 activation domain (AD), respectively. For each pair of AtNF-YB/AtNF-YC constructs, the Yeast Two-Hybrid interactions were tested in both configurations, to minimize the possibility of false positive and negative results. For both *NF-Y* gene families, we used the full length cDNAs corresponding to all *AtNF-YB* and *AtNF-YC* genes previously classified [9]. Three readouts were considered: His, Ade and LacZ, each driven by a different promoter under the control of the GAL4 responsive elements. Figure 1A shows the results of the different combinations with AtNF-YCs fused to the GAL4 DBD, and AtNF-YBs to GAL4 AD. On the other hand, Figure 1B shows the result obtained with AtNF-YCs fused to the GAL4 activation domain and AtNF-YBs to GAL4 DBD. Note that, in both cases, 3-AT was added to the yeast medium to minimize the growth due to self-activation. A first result is that the vast majority of the NF-YB and NF-YC family members can interact with each other in this *in vivo* assay. The only exception to this general observation is LEC1/AtNF-YB9, which does not interact significantly with any of the AtNF-YCs, in both configurations (Fig. 1), except for a suboptimal interaction with AtNF-YC3 and only with the AD configuration (Fig. 1A). A weaker interaction can be observed between specific pairs, like AtNF-YB2/AtNF-YC6 and AtNF-YB3/AtNF-YC7, in both configurations. Other pairs with suboptimal affinity are AtNF-YB2/AtNF-YC2, AtNF-YB3/AtNF-YC2, AtNF-YB3/AtNF-YC6, AtNF-YB4/AtNF-YC7 and AtNF-YC3/AtNF-YB10 (Fig. 1B). To further confirm these interactions and better quantify their strength, liquid Y2H Assays were performed by measuring β-GAL activity under conditions of exponential growth. For the liquid assay, we used the AtNF-YB (DBD) and AtNF-YC (AD) configurations shown in Figure 1B. The results of these experiments are shown in Figure 2. As previously determined by in plate assays, the liquid assay confirmed that LEC1/AtNF-YB9 (DBD) does not significantly interact above background levels with any AtNF-YC subunits. The liquid assay confirmed the weak interactions detected by the in plate assay. Furthermore, it was possible to detect a couple of additional weak interactions between AtNF-YB7/AtNF-YC6 and AtNF-YB10/AtNF-YC6. On the other hand, AtNF-YB1, AtNF-YB5 and AtNF-YB6, and to a lesser degree AtNF-YB2, showed robust interactions with all AtNF-YC family members. Overall, this set of experiments indicate that the vast majority of the HFD combinations heterodimerize, with few very specific exceptions.

### *In vitro* analysis

**Figure 3:**
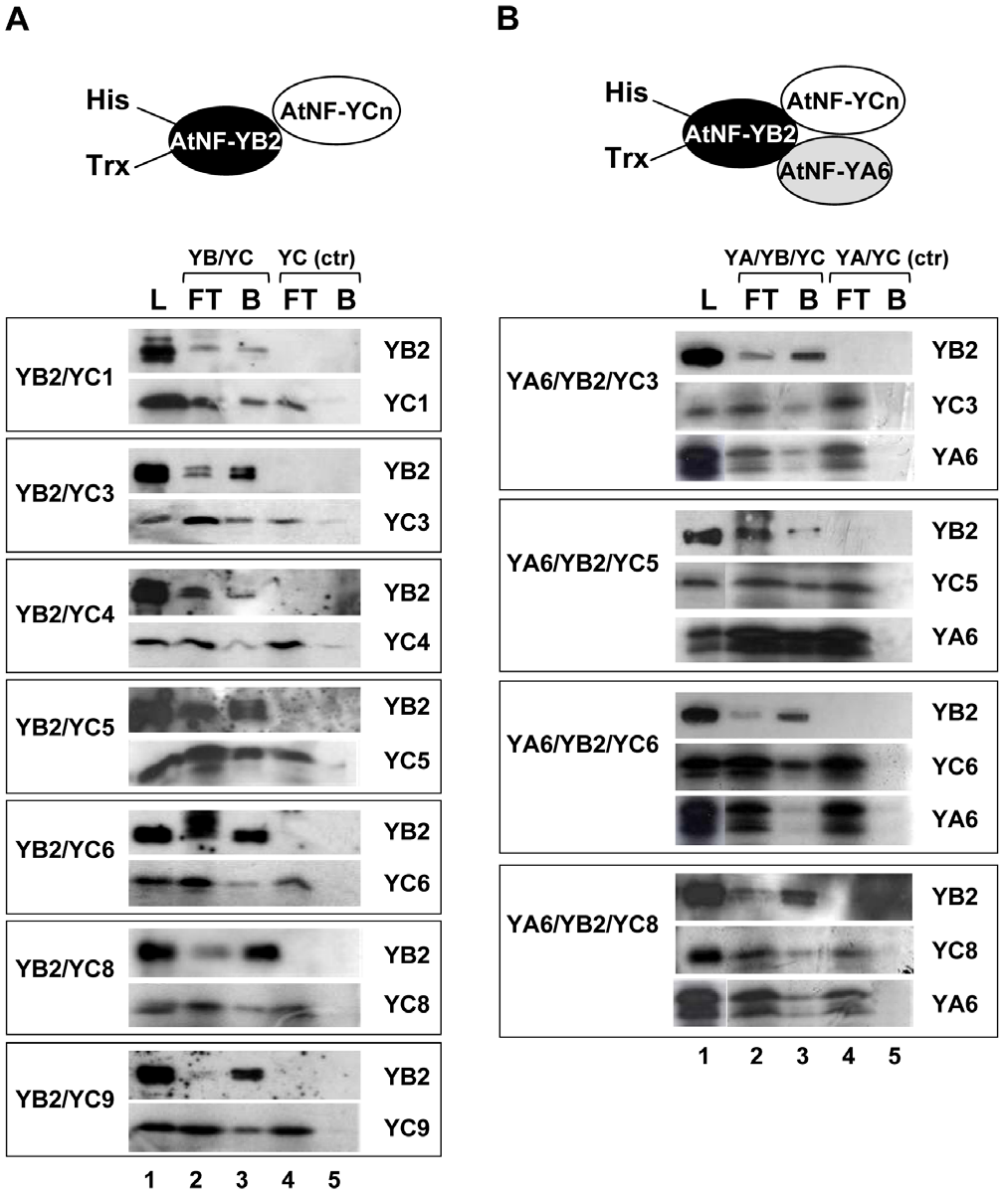
AtNF-Y Subunits interactions *in vitro*. **A**. The indicated labelled, TnT produced NF-YCs were assayed in affinity assays with recombinant AtNF-YB2 containing an His-tag. Load (L), flow-.through (FT) and bound (B) fractions of NTA Nickel columns, with (Lanes 2 and 3) and without (Lanes 4 and 5) His-AtNF-YB2 were run on SDS-PAGE gels and labelled proteins were revealed by autoradiography. **B**. Same as A, except that labelled, TnT produced AtNF-YA6 was added to the load fraction.

The negativity of LEC1/AtNF-YB9, unable to interact with any AtNF-YC, and the positivity of L1L/NF-YB6, which binds to all partners, are not expected. To substantiate the Y2H assays, we produced and purified recombinant proteins, as well as *in vitro* produced proteins by transcription and translation [TnT] of different subunits (Fig. S1). We chose AtNF-YB2 and AtNF-YA6 because they are rather “conventional” structure-wise when compared to the mammalian homologues. AtNF-YC were mixed with an excess of His-tagged recombinant AtNF-YB2 and loaded on NTA-Nickel columns. Figure 3 shows the results of such experiments. As expected, control columns did not retain any AtNF-YC subunit in the bound fractions in the absence of AtNF-YB2 (Fig. 3A and 3B, lanes 5). On the other hand, all AtNF-YCs were bound, with varying degrees of efficiency, in the presence of AtNF-YB2 (Fig. 3A and 3B, lanes 3), or L1L/AtNF-YB6 (Data not shown). While this assay is not quantitative, it does confirm that the two AtNF-YBs are able to retain on the column all AtNF-YCs, consistent with the results obtained by the Y2H assay. In the same assay, AtNF-YA6 was also retained with different AtNF-YC combinations when His-tagged AtNF-YB2 was added (Fig. 3B), indicating that interactions are observed in the presence of the three subunits.

**Figure 4:**
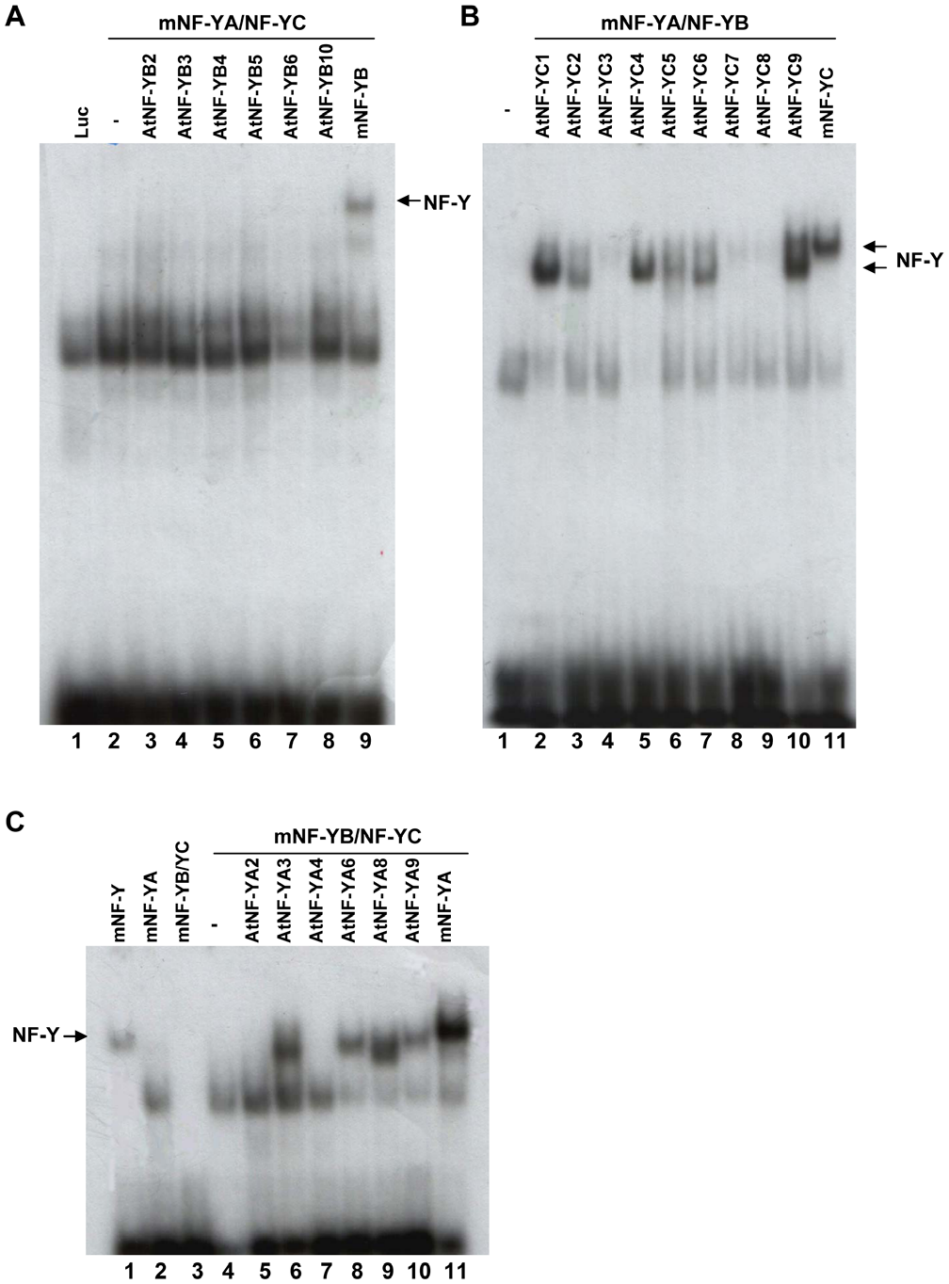
EMSAs of AtNF-Y subunits with mouse NF-Y. **A**.Electrophoretic Mobility Shift Assay of the indicated AtNF-YB with recombinant mouse NF-YA and NF-YC using a labeled CCAAT-containing oligonucleotide. **B**. Same as A, except that At NF-YCs were used with recombinant mouse NF-YA and NF-YB. **C**. Same as A, except that AtNF-YA were used with recombinant mouse NF-YB and NF-YC. The migration of the mouse NF-Y complex is indicated.

Having shown that most HFD subunits are able to interact both *in vivo* and *in vitro*, the next relevant question concerns the affinity of combinations for the CCAAT box. To answer this question, recombinant proteins were produced by TnT and used in EMSAs with a consensus, high affinity NF-Y oligonucleotide [1]. In Figure 4, several members of the *Arabidopsis* subunits were first assayed in the presence of the mouse NF-YA/NF-YC heterodimer. As negative controls we used the mouse dimeric combinations alone (Fig. 4A lane 2, Fig. 4B lane 1 and Fig. 4C lane 4). *In vitro* transcribed and translated Luciferase was added to the same mouse dimers as an additional negative control (Fig. 4A, lane 1). Recombinant mouse NF-YA alone was also used as negative control (Fig. 4C, lane 2). Positive controls were the mouse recombinant NF-Y trimer (Fig. 4C, lane 1), and single mouse NF-Y subunits added to the corresponding mouse dimeric combinations: NF-YB to NF-YA/NF-YC (Fig. 4A, lane 9), NF-YC to NF-YA/NF-YB (Fig. 4B, lane 11) and NF-YA to NF-YB/NF-YC (Fig. 4C, lane 11). Surprisingly, none of the AtNF-YBs added to the mouse NF-YA/NF-YC led to the formation of a complex with an electrophoretic activity different from the negative controls (Fig. 4A). In the case of the AtNF-YCs, instead, all subunits generated a discrete band with mouse NF-YA/NF-YB, with mobility somewhat similar to that of mouse NF-Y: the bands were weak for AtNF-YC3, AtNF-YC7 and AtNF-YC8, but quite robust for the other six AtNF-YCs tested. For AtNF-YAs, AtNF-YA2 and AtNF-YA4 were negative, whereas AtNF-YA3, AtNF-YA6, AtNF-YA8 and AtNF-YA9 were all capable of generating bands with mobilities similar to mouse NF-Y. These results indicate that the majority of the AtNF-YA and AtNF-YC members behave as canonical NF-Ys, as they associate with mouse subunits and bind to the CCAAT box.

**Figure 5:**
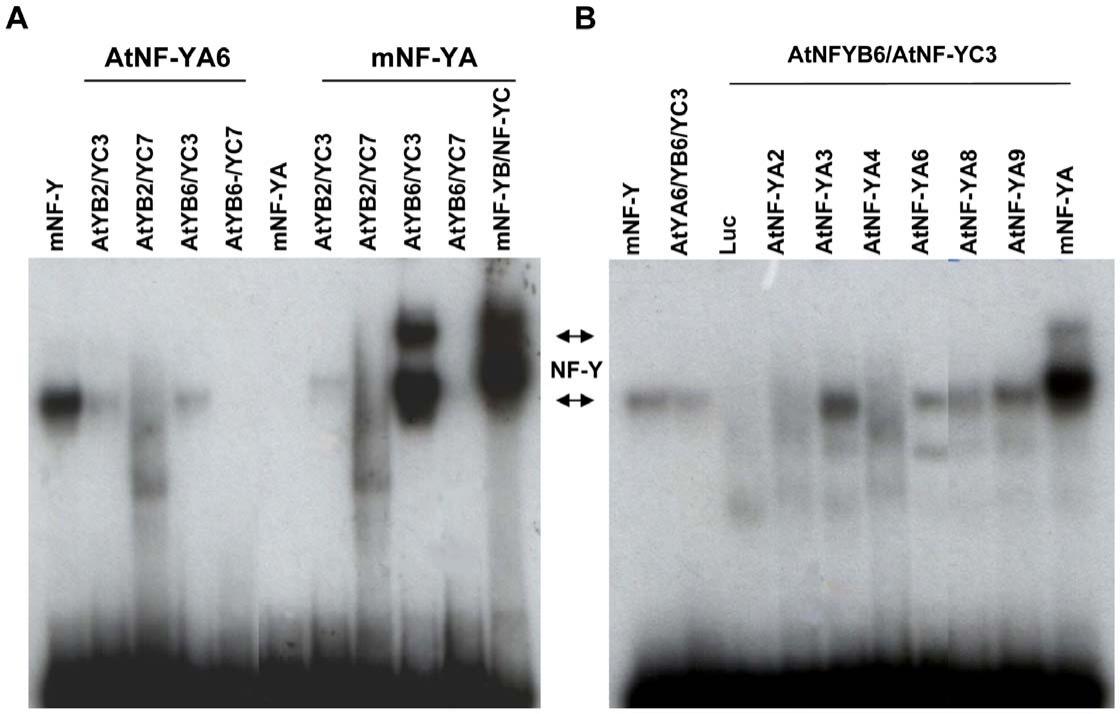
EMSAs of At NF-Y subunits. Electrophoretic Mobility Shift Assay of the indicated AtNF-Y subunits with a labelled CCAAT-containing oligonucleotide.

The negativity of the AtNF-YBs in the TnT-EMSA assays (Fig. 4A) was troubling: therefore, we decided to investigate whether this was an artefact due to the use of mouse recombinant NF-YA and NF-YC subunits and/or to the TnT system used. First, we selected two AtNF-YBs -AtNF-YB2 and L1L/AtNF-YB6- which are proficient in interactions with all AtNF-YCs according to the Y2H assay. We produced and purified single His-tagged AtNF-YB2 and AtNF-YB6 recombinant proteins in *E. coli*, together with two AtNF-YC subunits, namely AtNF-YC3 and AtNF-YC7 (Fig. S1). The choice of these members were driven by two types of considerations, the first being expression patterns, the second relatedness to mouse subunits: AtNF-YB2 and AtNF-YC3 are the most ubiquitously expressed and less “variant”, whereas L1L/AtNF-YB6 and AtNF-YC7 are strictly tissue-specific and the most deviant. The HFD proteins were found in inclusion bodies, as expected, denatured and efficiently renatured when mixed together [35], [36]. In one set of experiments, to the *Arabidopsis* NF-YB/NF-YC dimers we added recombinant AtNF-YA6, one of the AtNF-YAs positive in the EMSAs with mouse subunits (Fig. 5A). The *Arabidopsis* NF-YB/NF-YC dimers were also added to recombinant mouse NF-YA (Fig. 5A). AtNF-YA6 is able to generate NF-Y-like bands when AtNF-YC3 dimerized with either AtNF-YB2 or AtNF-YB6; the AtNF-YC7 combinations, on the other hand, yielded either no band or a smeary pattern. The same was essentially observed with mouse NF-YA (Fig. 5A), except that the AtNF-YB6/AtNF-YC3 combination was more efficient in binding, paralleling the efficiency of the mouse NF-Y trimer. The difference in mobilities of At-NF-YA6 and mouse NF-YA complexes are visible and most likely due to the different molecular mass of these two NF-YA proteins (308 and 347 residues, respectively). Again, the AtNF-YC7 combinations gave no band or a smear, indicating that heterotrimers with this subunit are very inefficient in CCAAT-binding. We decided to further dissect the DNA-binding activity of this heterodimer in the presence of other AtNF-YA family members: Figure 5B shows that an NF-Y complex was obtained with AtNF-YA3, AtNF-YA6, AtNF-YA8 and AtNF-YA9. Taken together, the results of Figure 5 are consistent with the set of experiments previously performed by using mouse recombinant subunits (Fig. 4). These data indicate that AtNF-YA2 and AtNF-YA4 are either incapable to associate to AtNF-YB6/AtNF-YC3 -and mouse HFD dimers- or to bind to DNA.

**Figure 6:**
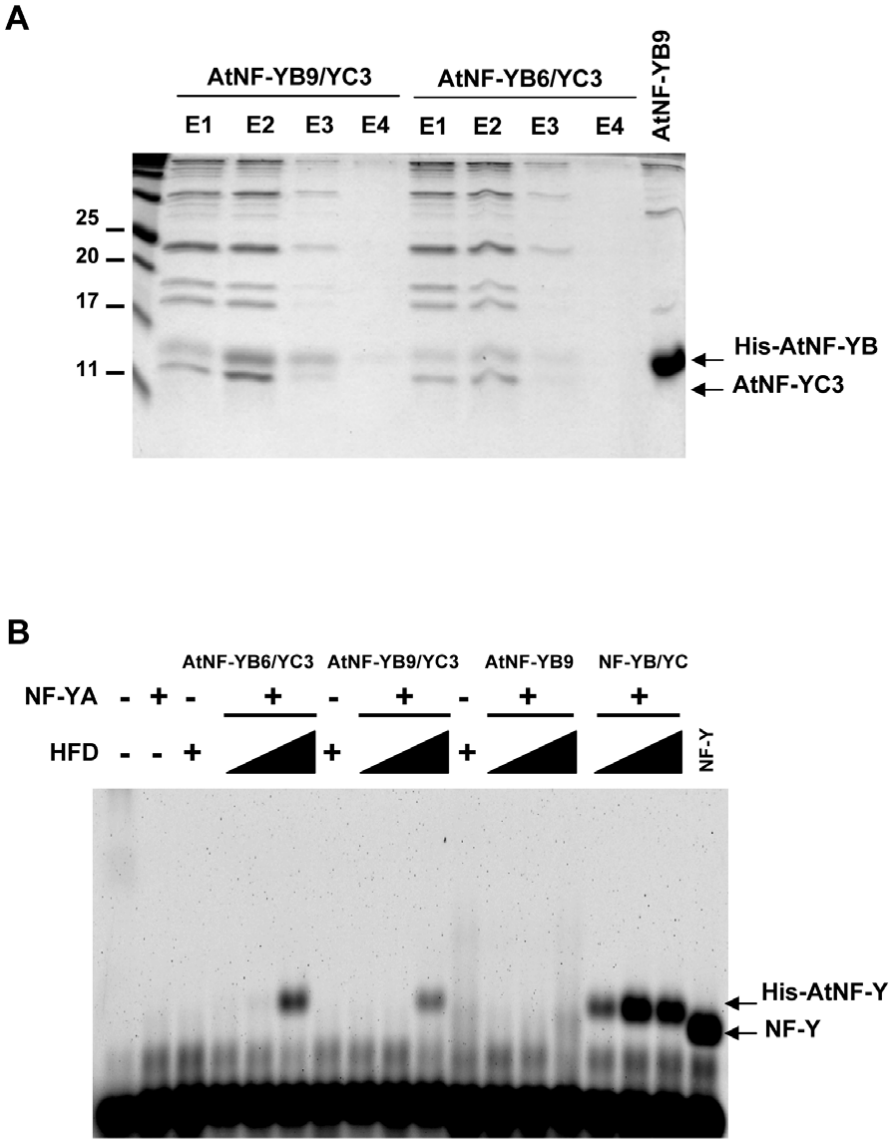
*E. coli* co-expression of LEC1/AtNF-YB9 with AtNF-YC3 allows functional heterodimerization, heterotrimerization and CCAAT-binding. **A**. Purification of soluble LEC1/AtNF-YB9 or L1L/AtNF-YB6 HFD heterodimers by co-expression with AtNF-YC3. Nickel-affinity purification elution profiles obtained from soluble fractions of 6His-LEC1/AtNF-YB9 or 6His-L1L/AtNF-YB6 with AtNF-YC3. Equal volumes of indicated elution fractions (E) in 100 mM Imidazole of LEC1/AtNF-YB9 or L1L/AtNF-YB6 with AtNF-YC3 were analysed by SDS-PAGE and Coomassie staining. E2, were dialysed and used in Agarose gel non-radioactive EMSAs shown in (B). **B**. Fluorescence agarose gel EMSAs of trimer reconstitution with mouse NF-YA. 5′-Cy5 labeled CCAAT oligonucleotide probe was incubated with increasing amounts of the indicated 6His-tagged HFD dimers isolated by Ni-affinity purification, or mouse 6His-NF-YB/NF-YC as positive control, in the presence, or absence, of purified mouse NF-YA. Purified (untagged) mouse NF-Y trimer was used as a reference for NF-Y complex migration.

L1L/AtNF-YB6 and LEC1/AtNF-YB9 belong to the same clade and are genetically linked. Having shown that L1L/AtNF-YB6 is capable to heterotrimerize and bind to CCAAT, we wondered whether the lack of heterodimerization of LEC1/AtNF-YB9 was due to some artefacts of the Y2H system. We decided to use an *E. coli* coexpression system in which the HFDs of either protein was coexpressed with the HFD of AtNF-YC3: Figure 6A shows that both heterodimers are produced and purified from soluble bacterial extracts. The copurification of (untagged) AtNF-YC3 with the His-tagged AtNF-YBs is a clear sign of heterodimerization. Surprisingly, when we expressed LEC1/AtNF-YB9 alone, rather than being confined to inclusion bodies, the protein was very efficiently produced in a soluble form, which is very unusual for HFD proteins. Next, we performed EMSAs with a CCAAT oligonucleotide, using the two heterodimers and mouse NF-YA: Figure 6B shows that both gave shifted bands, with mobilities similar to NF-Y. The affinities were lower with respect to the mouse NF-YB/NF-YC used as positive control, but similar among them. Note that in this particular experiments, we used the minimal heterotrimerization/DNA binding domain constructs consisting of the evolutionarily conserved regions of each subunit [3], with a 31 bp Cy5-labelled probe in Agarose-EMSA, which resulted in faster DNA-protein complexes. AtNF-YB9 alone did not show any DNA binding. Taken together, these data prove that LEC1/AtNF-YB9 can heterodimerize, trimerize with NF-YA and bind to CCAAT as efficiently as its closest relative, L1L/AtNF-YB6.

## Discussion

One of the most pressing questions in biology is to understand the interactions of transcription factors among each other and with their natural DNA targets. As they have often evolved in complex families, whose members share some common features and diverge in others, the intricacies of the role of each member needs to be clarified. This is particularly challenging in plants, where genes encoding TFs have expanded to amazing numbers. One such example is NF-Y, the heteromeric CCAAT-binding protein, whose subunits are encoded by single copy genes in most eukaryotes, including mammals [2], while in *Arabidopsis* and other plants each is represented by large families. We began a systematic investigation of the interactions among 24 AtNF-Y subunits, by using Y2H *in vivo* and *in vitro* assays. Some of our data, notably those on LEC1/AtNF-YB9, indicate that negative results of Y2H assays should be confirmed by independent biochemical means, before interactions can be ruled out.

### Dimerization

By and large, the *Arabidopsis* NF-Y HFD subunits -AtNF-YBs and AtNF-YCs- are able to heterodimerize, whether by Y2H assays or by *in vitro* interactions. These results are not surprising, based on considerations made on the crystallographic structure of the mouse NF-YB/NF-YC dimer [3], and of similar HFD dimers, including H2A/H2B [35]. These analyses have revealed that the α2 helix of the HFD is the core of the dimerization surface, thanks primarily to hydrophobic contacts. Another region of importance is the α1 helix, stabilized by hydrophobic interactions that are stacked against a conserved tryptophan at position 85 of α2 helix of NF-YC. Essentially all AtNF-YBs and AtNF-YCs have hydrophobic residues at appropriate positions, thus the widespread heterodimerizations we observed came to modest surprise. With respect to the Y2H experiments reported by Hackenberg et al. [34], as well as previous data [13], [31], we note the following differences.

- Some clear preferences for heterodimer formation between specific AtNF-YBs and AtNF-YCs were observed in the present and in the Hackerberg studies: only AtNF-YC6 and AtNF-YC7 show a marked preference for selected AtNF-YB subunits, in our study; all AtNF-YCs, except AtNF-YC3 and AtNF-YC9, have clear preferences in the Hackenberg data. On the AtNF-YBs side, AtNF-YB2, AtNF-YB3, AtNF-YB4, AtNF-YB7 and AtNF-YB10 show reduced affinity for one, or sometimes two AtNF-YCs: the same applies in the reported study. Even with our knowledge of the structure, it is quite difficult to rationalize these preferences, which seems quantitative more than qualitative.
- In the Hackenberg study, AtNF-YB11, B12, B13 and AtNF-YC10, 11, 13 are very selective, with a tendence to heterodimerize among them: the likeliest explanation is that these are not true NF-Y subunits, but rather resemble to other H2A/H2B-likes [KT, CT, RM, in preparation]: it should be remembered, in fact, that the TBP/TATA-binding NC2α/NC2β and the chromatin remodeling and DNA-Polymerase e subunits Chrac15/Dpb3/Dpb4 share extended conservation and have highly similar structures [2], [37], [39]. We have previously reported that mammalian NF-YB/NF-YC do not cross heterodimerize with NC2 subunits [38], and there are structural reasons for this [3].
- Kumimoto et al. have shown that AtNF-YB2 and B3 interact strongly with AtNF-YC3, C4 and C9 in Y2H and in genetic terms [31], which is in line with our data, but not with the Hackerberg study, in which they lack AtNF-YC4 binding. In rice, the homologue of AtNF-YB2 (OsHAP3A) interacts in Y2H with all OsHAP5s, including the homologue of AtNF-YC4, except for homologues of AtNF-YC2 and C3 [13].
- AtNF-YC5, AtNF-YC7 and AtNF-YC8, which belongs to a common clade and are the most tissue-restricted members of the AtNF-YC [8], were negative for AtNF-YB heterodimerization in the Hackenberg et al., study, the former in both AD and DB combinations, the latters in one. In our data, these AtNF-YCs were generally positive for all AtNF-YBs, except AtNF-YC7, negative with AtNF-YB3 and AtNF-YB4.
- LEC1/AtNF-YB9 showed a dual behaviour in our hands: no interactions with AtNF-YCs in the Y2H assays, yet efficient heterodimerization with AtNF-YC3 by co-expression of the two proteins in *E*. *coli*. In the Hackenberg study, LEC1/AtNF-YB9 was positive with most AtNF-YCs; moreover, the carrot homologue of LEC1 was able to bind DNA *in vitro* with two NF-YC homologues [33]. Thus, in this specific case, our Y2H was clearly misleading.

All in all, different Y2H data show some discrepancies, most likely due to technicalities in the expression vectors, yeast productions or activation assays. We also have to bear in mind that yeast possesses endogenous HAPs (as well as NC2 and Dpb3/4), indeed shown to interact with some of the plant members [30], thus possibly influencing the results of such assays. Our experiments with LEC1/AtNF-YB9 illustrates the dangers of relying only on this assay in the case of negative results.

We are intrigued by the unusual capacity of LEC1/AtNF-YB9 to form homodimers and remain soluble in bacteria: to the best of our knowledge, this is unique among HFDs, which are normally found as inactive, precipitated proteins in inclusion bodies, when not overexpressed with the appropriate partner [36]. This brings up the question of whether LEC1 homodimers are formed in plants. We found that they do not bind DNA, most likely because of lack of interactions with NF-YA, which absolutely requires NF-YC. It is possible that there is regulation of homo- to heterodimer formation: for example, post-translational modifications (PTMs), not performed in bacteria, could be required to render the HFD prone to heterodimerization: these are histone-like proteins, and histones are crucially controlled by a wealth of PTMs, and we have recently obtained evidence that mouse NF-YB is modified *a-la* H2B (RM, in preparation).

LEC1/AtNF-YB9 and L1L/AtNF-YB6 are capable to efficiently heterodimerize with AtNF-YC3, trimerize and bind to DNA, and the latter also with all AtNF-YC partners in Y2H assays. These data fits with genetic experiments, which established that L1L complements the LEC1 mutants, and in domain swapping experiments with other AtNF-YBs, the B domain-corresponding to the HFD [40]- is required for complementation. In addition to the AtNF-YC and AtNF-YA partners, LEC1 and L1L could exert their roles through interacting proteins, such as MADS box OsMADS6 and OsMADS18 [41], Pirin1, an iron-containing member of the cupin superfamily involved in a pathway leading to an ABA-mediated delay in seed germination [24]. Additional proteins interacting with AtNF-Ys are bZIP67, interacting with AtNF-YC2 in the regulation of *CRUCIFERIN C* [*CRC*] and *SUCROSE SYNTHASE2* [*SUS2*] in *Arabidopsis* protoplasts [21], and, most importantly, CONSTANS and CONSTANS-like proteins in *Arabidopsis* and tomato [42], [43] involved in determining the proper flowering timing with specific members of AtNF-YBs and AtNF-YCs [31], [44].

### DNA-binding

The formation of NF-Y heterotrimers was tested with selected AtNF-YB/AtNF-YC HFD dimers. While the HFD dimer contributes substantially to DNA-binding, mostly through α1 helices, L1 and L2 loops, the subunit that confers the sequence-specificity is NF-YA. On the HFD side, the heterotrimerization surface relies in selected residues in the α2 helix of NF-YB and in the αC helix of NF-YC. The E90 and E98 of mouse NF-YB, important for NF-YA binding [45], are conserved in all AtNF-YBs [8]–[10], [34]. The αC helix of AtNF-YC, on the other hand, shows differences in at least three members: AtNF-YC5 possesses an R at position 109 of mouse NF-YC, instead of an hydrophobic residue; AtNF-YC8 has two Aspartates at position 111 and 112, instead of hydrophobics, together with Isoleucine at position 113, instead of the helix capping Proline [3]; finally, AtNF-YC7 has a four aminoacids addition in the α3 helix, which extends it for an additional turn, hence displacing the LC domain and αC helix from their natural positions. Not coincidentally, these three members were not proficient in DNA-binding in our assays. Although the interaction with AtNF-YA6 appears to be visible with recombinant proteins, it remains to be seen whether other residues directly contacting DNA in L1 and L2 loops (N86 in AtNF-YC5 and G113 in AtNF-YC7, instead of a conserved Lysine) might explain the decrease in DNA affinity of this group of AtNF-YCs.

It was initially troubling to obtain negative results in EMSAs with the TnT-produced AtNF-YBs, but this was most likely due to technical problems of the translation extract, possibly inhibiting trimerization, or production of inactive AtNF-YBs in the absence of coexpression of AtNF-YCs: in fact, recombinant AtNF-YBs produced from *E. coli*, including the divergent LEC1/AtNF-YB9 and L1L/AtNF-YB6, were positive in DNA-binding. Interestingly, mutation of an Aspartate at position 55 of LEC1 is sufficient to abrogate LEC1 function *in vivo* [17]. D55 is located at the beginning of the α2 Helix, in a region that lies on the surface of the dimer: most other *Arabidopsis* and mammalian NF-YBs have a Lysine, conserved in H2B, and predicted to be involved in protein-DNA interactions [3]. L1L/AtNF-YB6 also has an Aspartate at this position, which might be considered as a “signature” for this subfamily: the change might decrease affinity for DNA, but an important result in our study is that it certainly does not abolish it: in essence, no AtNF-YB is “deviant” enough to have lost the DNA-binding capacity.

On the NF-YA side, the evolutionarily conserved domain is responsible for trimerization and CCAAT-binding. Protein-protein interaction assays and EMSAs indicate that the majority of AtNF-YAs are able to interact with AtNF-YB2/AtNF-YC3 and L1L/AtNF-YB6/AtNF-YC3. Indeed, they are quite proficient in association to the mouse NF-YB/NF-YC dimer. In particular, the AtNF-YA6 shows robust CCAAT binding, which strongly suggests that AtNF-YA5, not tested here, behaves similarly: the two belong to a common clade, and the DNA-binding subdomain is absolutely conserved. AtNF-YA5 is so far the only AtNF-YA for which genetic experiments were reported: mutation causes drought stress and overexpression drought resistance [25]; our data suggest that the mechanisms are related to prototypical CCAAT-binding.

Only AtNF-YA2 and AtNF-YA4 were negative, suggesting that they are either incapable to trimerize or bind DNA. Note that AtNF-YA7 and AtNF-YA10 not tested here might behave similarly, since the residues required for subunits interactions and DNA-binding are identical to AtNF-YA4 and AtNF-YA2, respectively. Several papers described two separate 20 aminoacid stretches as required for subunits interactions and DNA-binding [46]–[48]. Detailed mutagenesis of the mouse and yeast subunits pinpointed several aminoacids necessary for the two functions. In the subunits interaction domain, no dramatic changes are observed, and indeed important residues are conserved in AtNF-YA2 and AtNF-YA4, with the notable exception of R273 (mouse), which is G147 in AtNF-YA2 and G137 in AtNF-YA4: potentially, this could affect trimerization, since an R to G mutation in yeast HAP2 does decrease the efficiency of HFD association significantly [46]. We note, however, that in none of the other AtNF-YAs, nor in most other plant NF-YA genes, there is an Arginine at this position: in proficient members of the family tested here, an Alanine is present. Most importantly, AtNF-YA2 and AtNF-YA4 were previously tested for heterotrimerization, and indeed showed efficient association with HFDs [34]: in all likelyhood, therefore, they have decreased DNA-binding affinity, despite an overall conservation of key DNA-binding residues. Can we take these data as an indication that some of the AtNF-YAs have lost the capacity to bind DNA? If it is indeed so, what might be their function? The most obvious answer is that if they do bind NF-YB/NF-YC dimmer, they might act as Dominant Negative in terms of CCAAT binding: indeed, introduction of mutations in the DNA-binding subdomain of mouse NF-YA transforms it into a DN protein ([1] and References therein).

The alternative, more appealing possibility to explain these results is that trimers with these subunits have subtly changed sequence-specificity. Residues that are variant in these genes, such as C176 in AtNF-YA4 -a Serine in the other AtNF-YAs- and H178 in AtNF-YA2 -a Glutamate in the other AtNF-YAs- or the longer linker of AtNF-YA2 might account for this. Bioinformatic analysis performed in our lab on human genome-wide data has established that the NF-Y consensus, even in mammals, can, moderately, deviate from a perfect pentanucleotide CCAAT, provided that additional flanking nucleotides are present [2]: indeed, some 30% of NF-Y bound *in vivo* in human cells show a deviation of one nucleotide of the core CCAAT sequence. It seems reasonable therefore to postulate that subclasses of AtNF-YAs might bind variant versions of the CCAAT box: this hypothesis can be tested more thoroughly by the biochemical assays we set up with recombinant proteins, as we have started to do here. Even so, rationalization and full understanding of the molecular details of the enormous combinatorial possibilities of plant NF-Ys will have to ultimately await crystallization of NF-Y/CCAAT complexes.

## Materials and Methods

### Yeast strains and plasmid construction

The cDNAs corresponding to each AtNF-Y subunit used in the Yeast-Two Hybrid assay, were amplified from *Arabidopsis* cDNA libraries using gene specific primers containing the attB1 and attB2 sequences for homologous recombination and subsequently cloned into pDONOR201 vector (Life Technology). *AtNF-YB* and *AtNF-C* coding sequences in pDONOR201 were subsequently cloned in the GAL4 Gateway vector system: pDEST32 for DNA binding domain fusions (pDBD) and pDEST22 for activation domain fusions (pAD). The pDEST32 and pDEST22 vectors were transformed into *Saccharomyces cerevisiae* strain PJ69-4A (trp1-901 leu2-3, 112 ura3-52 his3-200 gal4Δ gal80Δ LYS2::GAL1-HIS3 GAL2-ADE2 met2::GAL7-lacZ) [49]. Yeast Two-Hybrid assay was performed as described below.

### Yeast Two-Hybrid (Y2H) analysis

Haploid Yeast α and A were transformed respectively with pBD and pAD vector constructs using the lithium acetate method [47] and selected on Yeast Synthetic Dropout [YSD] medium lacking Leu and Trp, respectively. Yeast carrying pBD vectors were tested for autoactivation on selective medium with 5-bromo-4-chloro-3-indolyl-b-D-galactopyranoside (X-Gal), on medium lacking histidine and supplemented with different concentrations of 3-aminotriazole (0, 3, 5, 10, 25 and 50 mM) and on medium lacking adenine. Mating type α and A were mated and diploids selected on YSD medium lacking Leu and Trp.

Two-hybrid interactions were assayed on selective YSD medium lacking Leu, Trp, and Ade or His supplemented with 50 mM 3-aminotriazole. Selection was performed at 28°C for 4 days.

### Liquid Two-Hybrid Assay

Semi-quantitative assay for comparing the strength of AtNF-YB and AtNF-YC subunits interactions was performed by liquid LacZ assay. For the liquid assay, we used the AtNF-YB (DBD) and AtNF-YC (AD) configuration.

Yeast was inoculated in selective medium and grown for 8–9 h, then centrifuged at 4500 rpm for 5 min. Pelletted cells were resuspended in 5 ml of selective medium and grown O/N at 28°C. Cells were centrifuged at 4500 rpm for 5 min and the pellet was resuspended in 0.5 ml of cold water, centrifuged again for 30 sec at 14000 rpm. Pellet was resuspended in 250 µl of pre-cooled Breaking Buffer (100 mM Tris-HCl pH 8.0, 10% Glycerol, 1 mM Dithiothreitol, protease inhibitors) and frozen in liquid nitrogen. Unfrozen samples were subjected to 10 cycles of vortex/ice with glass beads, centrifuged at 14000 rpm for 10 min and supernatant has been recovered. Then 20 µl of protein extract were transferred to a 1.5 ml centrifuge tube and added with 800 µl of Z-Buffer 1× (60 mM NaH_2_PO_4_, 40 mM Na_2_HPO_4_ anhydrous, 10 mM KCl, 1 mM Mg2SO4, 50 mM β-Mercaptoethanol) and 200 µl ortho-Nitrophenyl-β-galactoside (ONPG) 4 mg/ml. The tube was incubated at 37°C until the solution became yellow, for a maximum of 45 min and the reaction was stopped adding 400 µl of 1.5 M Na_2_CO_3_. The samples were centrifuged for 30 sec at 13000 rpm and the optical density at 420 nm (OD_420_) was determined. Activity in Miller Units was calculated according to the formula (OD_420_ *1.4)/(0.0045*C*V*t) where C = concentration of protein extract (mg/ml); V = volume of protein extract (ml); t = time (min). Activity of AtNF-YB GAL4-DBD with GAL4-AD fused with no AtNF-YC subunit has been used as control.

### Production of recombinant AtNF-YB and generation of ^35^S-Labeled AtNF-YC

To examine the *in vitro* interaction between AtNF-YB and AtNF-YC subunits, His-tagged AtNF-YBs and ^35^S labelled AtNF-YC were produced and used for pull-down experiments.

Chemically competent *E. coli* BL21 cells were transformed by thermal shock with 100 ng of pET32A or pET32B, in which AtNF-YB coding sequences were cloned. Transformed cells were inoculated in LB broth (5 ml) with ampicillin (100 ng/ml) at 37°C for 16 h. An aliquot (3 ml) of this culture was inoculated in 200 ml of the same medium and let grow until an OD_600_ of 0.6 was reached. The expression of each protein was induced with IPTG (1 mM) for 3 h.

Cells were harvested by centrifugation at 6000 rpm for 10 min at 4°C and suspended in Sonication Buffer (300 mM KCl, 20 m M Tris-HCl pH 7.8, 0.05% NP40, 1 mM EDTA, 1 mM PMSF, 5 mM β-Mercaptoethanol) containing a cocktail of Protease inhibitors (12.5 mg/ml leupeptin, 5 mg/ml trypsin inhibitor, 5 mg/ml pepstatin, 10 mg/ml chymostatin). The cells were then thoroughly disrupted with a sonicator (10 cycles, 20 sec each). The samples were centrifuged at 23000 rpm at 4°C for 90 min to separate supernatant (SN) from inclusion bodies (IB). The SN and IB (Resuspended in 100 mM KCl, 20 mM Tris-HCl pH 7.8, 5 mM β-Mercaptoethanol, 1 mM PMSF, 6 M GnCl) were loaded onto Nichel-Agarose columns (Sigma). After thoroughly washing with Washing Buffer (20 mM Tris-HCl pH 7.8 10% glycerol, 300/1000/100 mM KCl), the proteins bound to the columns were eluted in Elution Buffer (2 mM Tris-HCl pH 7.8, 10% glycerol, 100 mM KCl, 1 mM PMSF, 5 mM β-Mercaptoethanol, 300 mM Imidazol). Finally, eluted fractions from SN and IB were subjected to dialysis to remove Imidazol.

AtNF-YC subunits, cloned in pCR4TOPO (Invitrogen), were synthesized and ^35^S-labeled by coupled transcription and translation in 25 µl of nuclease-treated rabbit reticulocyte lysate (TnT, Promega).

### His pull-down assay

His-tagged AtNF-YB recombinant proteins (500 ng) and 10 µl of AtNF-YCs produced by TnT were incubated together at 37°C for 30 min in 100 µl of NDB100 (20% glycerol, 100 mM KCl, 20 mM Tris-HCl pH 7.8, 0.5 mM EDTA, 5 mM β-Mercaptoethanol). After incubation, recombinant proteins were loaded onto a Nichel-Agarose column (Sigma), incubated for 3 h at 4°C, and then centrifuged at 4000 rpm for 1 min at 4°C to recover the “flow through” (FT). After washing 3 times, they were eluted (“bound”, B) with 30 µl of Elution Buffer (NDB100 containing 5 mM β-Mercaptoethanol, 0.25 M imidazole, PIC 1×). As negative controls, aliquots (10 µl) of the same AtNF-YC subunits producted by TnT were incubated with the Nichel-Agarose column. We did not observe any aspecifically bound AtNF-YC subunits in the negative controls performed in the absence of His-tagged AtNF-YBs. One third of FT and B samples were subjected to SDS-PAGE, transferred to a nitrocellulose membrane (150 mA/gel, 1.5 h), and analyzed by Western blotting using anti-His antibodies; the remaining two thirds of each sample were analysed by autoradiography to detect AtNF-YC subunits.

### HFD heterodimer Protein expression and purification

The 6His-AtNF-YB/AtNF-YC soluble HFD dimers were purified exploiting the T7-driven co-expression system described in [3], [50]. AtNF-YC3 (AA 55–148) (corresponding to the HFD region of mouse NF-YC AA 27–120) was subcloned in the pmncYC vector; LEC1/AtNF-YB9 (AA 56–148) or L1L/AtNF-YB6 (AA 26–118) subunits (corresponding to mouse NF-YB HDF AA 49–141) were subcloned in pET15b, resulting in 6His-N-terminal fusions. 6His-LEC1/AtNF-YB9, or 6His-L1L/AtNF-YB6, was expressed in *E. coli* BL21(DE3) together with, or not, AtNF-YC3, and purified by Ni-chelate affinity chromatography (HisSelect, SIGMA-Aldrich), as described in [3], in buffer A (10 mM Tris pH 8.0, 400 mM NaCl, 2 mM MgCl_2_, 5 mM imidazole), and eluted by subsequent additions of 1 bed volume of buffer B containing 100 mM Imidazole. Indicated 6His-HFD protein purification eluates were dialysed against buffer B (10 mM Tris pH 8.0, 400 mM NaCl, 2 mM DTT) containing 10% glycerol, and used in Fluorescensce Agarose gel EMSAs. The soluble NF-Y heterotrimeric subunit complex and 6His-NF-YA were produced as described in [50], and purified by Ni-chelate affinity chromatography (HisSelect, SIGMA-Aldrich) in buffer A, followed by thrombin cleavage of the NF-YA C-terminal His-tag, and gel filtration (GF) chromatography (HiLoad Superdex75, Amersham Pharmacia) in buffer B. GF fractions corresponding to the NF-Y heterotrimer, or the NF-YA isolated subunit, were collected, and used in Fluorescensce Agarose gel EMSAs, after addition of 10% glycerol for storage.

### Electrophoretic Mobility Shift Assays

For electrophoretic mobility shift assays ^32^P labelled fragments −10000 CPMs- are incubated in NF-Y Buffer (20 mM Hepes pH 7.9, 50 mM NaCl, 5% Glycerol, 5 mM MgCl_2_, 5 mM β-ME) with the recombinant proteins (1–5 ng), in a total volume of 10 µl; after incubation for 15′ at 20°C, we added 2 µl of 1× NF-Y buffer containing Bromophenol Blue and samples loaded on a 4.5% Polyacrylamide in 0.5× TBE. Gels were dried and exposed. For Fluorescence Agarose Gel EMSAs of Figure 6, heterotrimer formation and CCAAT-box DNA-binding of the 6His-AtNF-YB/AtNF-YC soluble dimers was assessed with Cy5-labeled oligos, by addition of GF purified mouse NF-YA (AA 233–303). Equal protein amounts of Ni-purified 6His-AtNF-YB/NF-YC HFD dimers (3, 6, or 9 ng/ul) were mixed in 15 µl reactions with the 5′-labeled 31 bp oligo probe derived from human HSP70 promoter CCAAT box sequence (Cy5-CTTCTGAGCCAATCACCGAGCTCGATGAGGC) in DNA binding mix (20 nM ds oligo, 20 mM Tris pH 7.5, 50 mM NaCl, 0.5 mM EDTA, 5 mM MgCl_2_, 2.5 mM DTT, 0.1 mg/ml BSA, 5% glycerol), in the presence of 40 nM NF-YA, where indicated. Ni-purified mouse 6His-NF-YB/NF-YC (1, 3, 6 ng), or GF purified NF-Y trimer (60 nM) were used a positive controls. After 30 min incubation at 23°C, binding reactions were loaded on a 2.5% agarose gel and separated by electrophoresis in 0.5× TBE. Fluorescence gel images were obtained with a Typhoon 8610 Variable Mode Imager (Molecular Dynamics).

## Supporting Information

Figure S1 **TnT and recombinant proteins production.** **A.** AtNF-YA, AtNF-YB and AtNF-YC subunits were synthesized and ^35^S-labeled by coupled transcription and translation in nuclease-treated rabbit reticulocyte lysate (TnT, Promega). **B.** His-tagged AtNF-YA6, AtNF-YB2 and AtNF-YB6, AtNF-YC3 and AtNF-YC7 have been produced in *E. Col*i and purified by Nichel-Agarose columns (Sigma). Load (L), flow-through (FT), wash (W) and eluted (E) fractions of NTA Nickel columns are shown.
(TIF)

## References

[1] Dolfini D, Zambelli F, Pavesi G, Mantovani R (2009) A perspective of promoter architecture from the CCAAT box. Cell Cycle 9: 4127–4237. 10.4161/cc.8.24.10240 19946211

[2] Dolfini D, Gatta R, Mantovani R (2012) NF-Y and the transcriptional activation of CCAAT promoters. Crit Rev Biochem Mol Biol 47: 29–49. 22050321 10.3109/10409238.2011.628970

[3] Romier C, Cocchiarella F, Mantovani R, Moras D (2003) The crystal structure of the NF-YB/NF-YC heterodimer gives insight into transcription regulation and DNA binding and bending by transcription factor NF-Y. J Biol Chem 278: 1336–1345. 12401788 10.1074/jbc.M209635200

[4] Forsburg SL, Guarente L (1989) Identification and characterization of HAP4: a third component of the CCAAT-bound HAP2/HAP3 heteromer. Genes Dev 3: 1166–1178. 2676721 10.1101/gad.3.8.1166

[5] McNabb DS, Tseng KA, Guarente L (1997) The *Saccharomyces cerevisiae* Hap5p homolog from fission yeast reveals two conserved domains that are essential for assembly of heterotetrameric CCAAT-binding factor. Mol Cell Biol 17: 7008–7018. 9372932 10.1128/mcb.17.12.7008 PMC232557

[6] Coustry F, Maity SN, Sinha S, de Crombrugghe B (1996) The transcriptional activity of the CCAAT-binding factor CBF is mediated by two distinct activation domains, one in the CBF-B subunit and the other in the CBF-C subunit. J Biol Chem 271: 14485–14491. 8662945 10.1074/jbc.271.24.14485

[7] de Silvio A, Imbriano C, Mantovani R (1999) Dissection of the NF-Y transcriptional activation potential. Nucleic Acids Res 27: 2578–2584. 10373572 10.1093/nar/27.13.2578 PMC148464

[8] Gusmaroli G, Tonelli C, Mantovani R (2001) Regulation of the CCAAT-binding NF-Y subunits in *Arabidopsis thaliana* . Gene 264: 173–185. 11250072 10.1016/s0378-1119(01)00323-7

[9] Gusmaroli G, Tonelli C, Mantovani R (2002) Regulation of novel members of the *Arabidopsis thaliana* CCAAT-binding nuclear factor Y subunits. Gene 283: 41–48. 11867211 10.1016/s0378-1119(01)00833-2

[10] Siefers N, Dang KK, Kumimoto RW, Bynum WE IV, Tayrose G, et al (2009) Tissue specific expression patterns of *Arabidopsis thaliana* NF-Y transcription factors suggest potential for extensive combinatorial complexity. Plant Physiol 149: 625–641. 19019982 10.1104/pp.108.130591 PMC2633833

[11] Yang J, Xie Z, Glover BJ (2005) Asymmetric evolution of duplicate genes encoding the CCAAT-binding factor NF-Y in plant genomes. New Phytol 165: 623–631. 15720672 10.1111/j.1469-8137.2004.01260.x

[12] Stephenson TJ, McIntyre CL, Collet C, Xue GP (2007) Genome-wide identification and expression analysis of the NF-Y family of transcription factors in *Triticum aestivum* . Plant Mol Biol 65: 77–92. 17598077 10.1007/s11103-007-9200-9

[13] Thirumurugan T, Ito Y, Kubo T, Serizawa A, Kurata N (2008) Identification, characterization and interaction of HAP family genes in rice. Mol Genet Genomics 279: 279–289. 18193457 10.1007/s00438-007-0312-3

[14] Xie Z, Li X, Glover BJ, Bai S, Rao GY, et al (2008) Duplication and functional diversification of HAP3 genes leading to the origin of the seed-developmental regulatory gene, LEAFY COTYLEDON1 (LEC1), in nonseed plant genomes. Mol Biol Evol 25: 1581–1592. 18453547 10.1093/molbev/msn105

[15] Cao S, Kumimoto RW, Siriwardana CL, Risinger JR, Holt BF III (2011) Identification and characterization of NF-Y transcription factor families in the monocot model plant *Brachypodium distachyon* . PLoS One 6: e21805. 21738795 10.1371/journal.pone.0021805 PMC3128097

[16] Lotan T, Ohto M, Yee MK, West MA, Lo R, et al (1998) *Arabidopsis* LEAFY COTILEDON1 is sufficient to induce embryo development in vegetative cells. Cell 93: 1195–1205. 9657152 10.1016/s0092-8674(00)81463-4

[17] Lee H, Fischer RL, Goldberg RB, Harada JJ (2003) *Arabidopsis* LEAFY COTYLEDON1 represents a functionally specialized subunit of the CCAAT binding transcription factor. Proc Natl Acad Sci USA 100: 2152–2156. 12578989 10.1073/pnas.0437909100 PMC149974

[18] Gaj MD, Zhang S, Harada JJ, Lemaux PG (2005) Leafy cotyledon genes are essential for induction of somatic embryogenesis of *Arabidopsis* . Planta 222: 977–988. 16034595 10.1007/s00425-005-0041-y

[19] Braybrook SA, Harada JJ (2008) LECs go crazy in *embryo* development. Trends Plant Sci 13: 624–30. 19010711 10.1016/j.tplants.2008.09.008

[20] Kwong RW, Bui AQ, Lee H, Kwong LW, Fischer RL, et al (2003) LEAFY COTYLEDON1-LIKE defines a class of regulators essential for *embryo* development. Plant Cell 15: 5–18. 12509518 10.1105/tpc.006973 PMC143447

[21] Yamamoto A, Kagaya Y, Toyoshima R, Kagaya M, Takeda S, et al (2009) *Arabidopsis* NF-YB subunits LEC1 and LEC1-LIKE activate transcription by interacting with seed-specific ABRE-binding factors. Plant J 58: 843–56. 19207209 10.1111/j.1365-313X.2009.03817.x

[22] Yazawa K, Takahata K, Kamada H (2004) Isolation of the gene encoding Carrot leafy cotyledon1 and expression analysis during somatic and zygotic embryogenesis. Plant Physiol Biochem 42: 215–223. 15051045 10.1016/j.plaphy.2003.12.003

[23] Alemanno L, Devic M, Niemenak N, Sanier C, Guilleminot J, et al (2008) Characterization of leafy cotyledon1-like during embryogenesis in *Theobroma cacao* L. Planta 227: 853–866. 18094994 10.1007/s00425-007-0662-4

[24] Warpeha KM, Upadhyay S, Yeh J, Adamiak J, Hawkins SI, et al (2007) The GCR1, GPA1, PRN1, NF-Y signal chain mediates both blue light and abscisic acid responses in *Arabidopsis* . Plant Physiol 143: 1590–1600. 17322342 10.1104/pp.106.089904 PMC1851835

[25] Li WX, Oono Y, Zhu J, He XJ, Wu JM, et al (2008) The *Arabidopsis* NFYA5 transcription factor is regulated transcriptionally and posttranscriptionally to promote drought resistance. Plant Cell 20: 2238–2251. 18682547 10.1105/tpc.108.059444 PMC2553615

[26] Nelson DE, Repetti PP, Adams TR, Creelman RA, Wu J, et al (2007) Plant nuclear factor Y (NF-Y) B subunits confer drought tolerance and lead to improved corn yields on water-limited acres. Proc Natl Acad Sci USA 104: 16450–16455. 17923671 10.1073/pnas.0707193104 PMC2034233

[27] Ito Y, Thirumurugan T, Serizawa A, Hiratsu K, Ohme-Takagi M, et al (2011) Aberrant vegetative and reproductive development by overexpression and lethality by silencing of OsHAP3E in rice. Plant Sci 181: 105–110. 21683874 10.1016/j.plantsci.2011.04.009

[28] Cai X, Ballif J, Endo S, Davis E, Liang M, et al (2007) A putative CCAAT-binding transcription factor is a regulator of flowering timing in *Arabidopsis* . Plant Physiol 145: 98–105. 17631525 10.1104/pp.107.102079 PMC1976580

[29] Chen NZ, Zhang XQ, Wei PC, Chen QJ, Ren F, et al (2007) AtHAP3b plays a crucial role in the regulation of flowering time in *Arabidopsis* during osmotic stress. J Biochem Mol Biol 40: 1083–1089. 18047807 10.5483/bmbrep.2007.40.6.1083

[30] Kumimoto RW, Adam L, Hymus GJ, Repetti PP, Reuber TL, et al (2008) The Nuclear Factor Y subunits NF-YB2 and NF-YB3 play additive roles in the promotion of flowering by inductive long-day photoperiods in *Arabidopsis* . Planta 228: 709–723. 18600346 10.1007/s00425-008-0773-6

[31] Kumimoto RW, Zhang Y, Siefers N, Holt BF III (2010) NF-YC3, NF-YC4 and NF-YC9 are required for CONSTANS-mediated, photoperiod-dependent flowering in *Arabidopsis thaliana* . Plant J 63: 379–391. 20487380 10.1111/j.1365-313X.2010.04247.x

[32] Combier JP, Frugier F, de Billy F, Boualem A, El-Yahyaoui F, et al (2006) MtHAP2-1 is a key transcriptional regulator of symbiotic nodule development regulated by microRNA169 in *Medicago truncatula* . Genes Dev 20: 3084–3088. 17114582 10.1101/gad.402806 PMC1635144

[33] Yazawa K, Kamada H (2007) Identification and characterization of carrot HAP factors that form a complex with the *embryo*-specific transcription factor C-LEC1. J Exp Bot 13: 3819–3828. 10.1093/jxb/erm238 18057048

[34] Hackenberg D, Wu Y, Voigt A, Adams R, Schramm P, et al (2012) Studies on Differential Nuclear Translocation Mechanism and Assembly of the Three Subunits of the *Arabidopsis thaliana* Transcription Factor NF-Y. Mol Plant In press. 10.1093/mp/ssr107 22199235

[35] Luger K, Mader AW, Richmond RK, Sargent DF, Richmond TJ (1997) Crystal structure of nucleosome core particle at 2.8A resolution. Nature 389: 251–260. 9305837 10.1038/38444

[36] Li XY, Mantovani R, Hooft van Huijsduijnen R, Andre I, Benoist C, et al (1992) Evolutionary variation of the CCAAT-binding transcription factor NF-Y. Nucleic Acids Res 20: 1087–1091. 1549471 10.1093/nar/20.5.1087 PMC312095

[37] Hartlepp KF, Fernández-Tornero C, Eberharter A, Grüne T, Müller CW, et al (2005) The histone fold subunits of Drosophila CHRAC facilitate nucleosome sliding through dynamic DNA interactions. Mol Cell Biol 25: 9886–9896. 16260604 10.1128/MCB.25.22.9886-9896.2005 PMC1280263

[38] Zemzoumi K, Frontini M, Bellorini M, Mantovani R (1999) NF-Y histone fold α1 helices help impart CCAAT specificity. J Mol Biol 286: 327–337. 9973554 10.1006/jmbi.1998.2496

[39] Kamada K, Shu F, Chen H, Malik S, Stelzer G, et al (2001) Crystal structure of negative cofactor 2 recognizing the TBP-DNA transcription complex. Cell 106: 71–81. 11461703 10.1016/s0092-8674(01)00417-2

[40] Masiero S, Imbriano C, Ravasio F, Favaro R, Pelucchi N, et al (2002) Ternary complex formation between MADS-box transcription factors and the histone fold protein NF-YB. J Biol Chem 277: 26429–26435. 11971906 10.1074/jbc.M202546200

[41] Wenkel S, Turck F, Singer K, Gissot L, Le Gourrierec J, et al (2006) CONSTANS and the CCAAT box binding complex share a functionally important domain and interact to regulate flowering of *Arabidopsis* . Plant Cell 18: 2971–2984. 17138697 10.1105/tpc.106.043299 PMC1693937

[42] Ben-Naim O, Eshed R, Parnis A, Teper-Bamnolker P, Shalit A, et al (2006) The CCAAT binding factor can mediate interactions between CONSTANS-like proteins and DNA. Plant J 46: 462–476. 16623906 10.1111/j.1365-313X.2006.02706.x

[43] Li C, Distelfeld A, Comis A, Dubcovsky J (2011) Wheat flowering repressor VRN2 and promoter CO2 compete for interactions with NUCLEAR FACTOR-Y complexes. Plant J 67: 763–773. 21554456 10.1111/j.1365-313X.2011.04630.x PMC4765905

[44] Sinha S, Kim IS, Sohn KY, deCrombrugghe B, Maity SN (1996) Three classes of mutations in the A subunit of the CCAAT-binding factor CBF delineate functional domains involved in the three-step assembly of the CBF-DNA complex. Mol Cell Biol 16: 328–337. 8524312 10.1128/mcb.16.1.328 PMC231007

[45] Xing Y, Fikes JD, Guarente L (1993) Mutations in yeast Hap/HAP3 define a hybrid CCAAT box binding domain. EMBO J 12: 4647–4655. 8223474 10.1002/j.1460-2075.1993.tb06153.x PMC413902

[46] Xing Y, Zhang S, Olesen JT, Rich A, Guarente L (1994) Subunit interaction in the CCAAT-binding heteromeric complex is mediated by a very short alpha-helix in HAP2. Proc Natl Acad Sci USA 91: 3009–3013. 8159696 10.1073/pnas.91.8.3009 PMC43504

[47] Mantovani R, Li XY, Pessara U, Hooft van Huijsduijnen R, Benoist C, et al (1994) Dominant negative analogs of NF-YA. J Biol Chem 269: 20340–20346. 8051128

[48] Gietz D, St Jean A, Woods RA, Schiestl RH (1992) Improved method for high efficiency transformation of intact yeast cells. Nucleic Acids Res 20: 1425. 1561104 10.1093/nar/20.6.1425 PMC312198

[49] James P, Halladay J, Craig EA (1996) Genomic libraries and a host strain designed for highly efficient two-hybrid selection in yeast. Genetics 144: 1425–1436. 8978031 10.1093/genetics/144.4.1425 PMC1207695

[50] Diebold ML, Fribourg S, Koch M, Metzger T, Romier C (2011) Deciphering correct strategies for multiprotein complex assembly by co-expression: application to complexes as large as the histone octamer. J Struct Biol 175: 178–188. 21320604 10.1016/j.jsb.2011.02.001

