# Supplementary figures and images for: Interactions and CCAAT-Binding of Arabidopsis thaliana NF-Y Subunits
Source: PLoS One. 2012 Aug 17;7(8):e42902. doi: 10.1371/journal.pone.0042902 (PMC3422339; doi:10.1371/journal.pone.0042902)

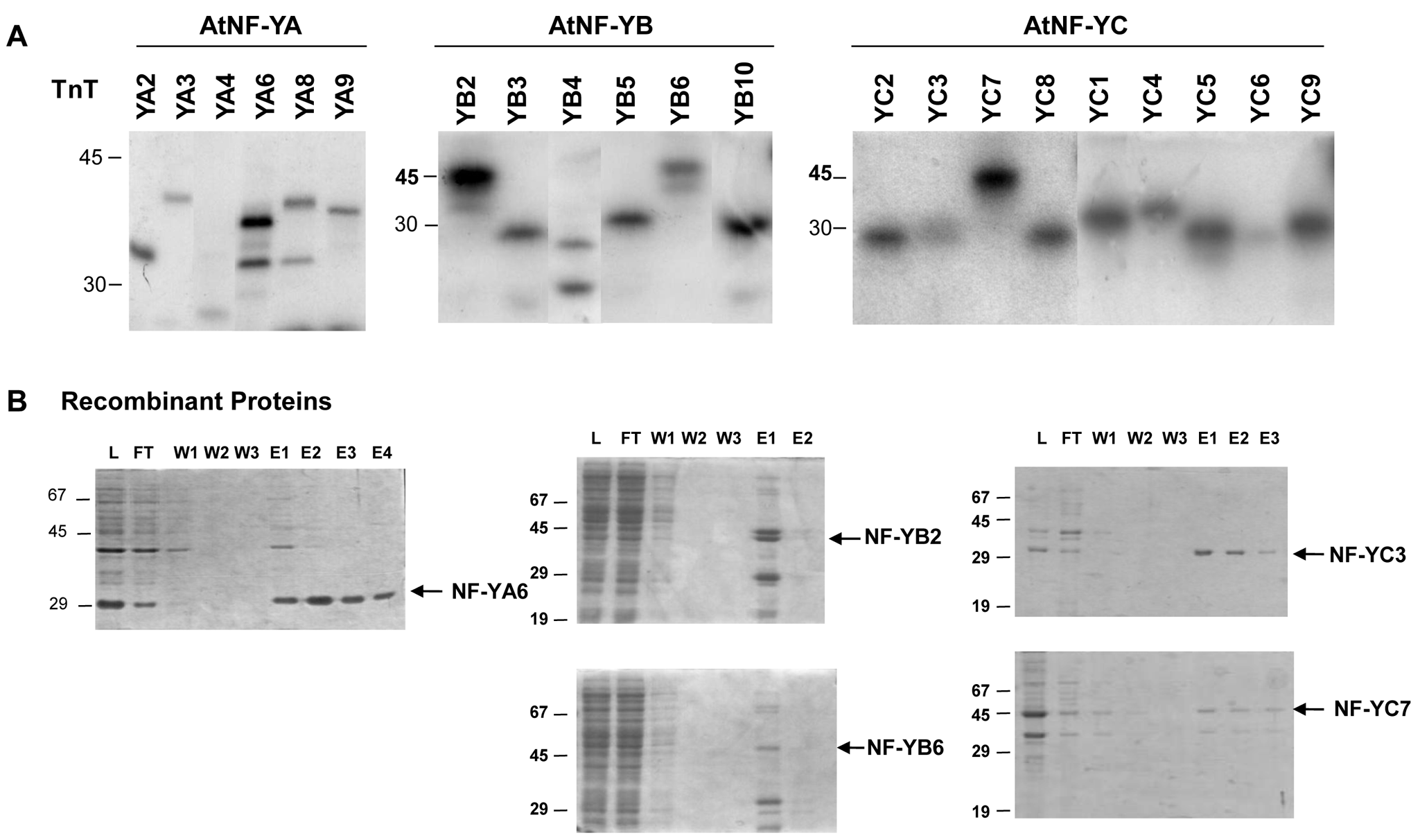

Supplement: Figure S1 — TnT and recombinant proteins production. A. AtNF-YA, AtNF-YB and AtNF-YC subunits were synthesized and 35S-labeled by coupled transcription and translation in nuclease-treated rabbit reticulocyte lysate (TnT, Promega). B. His-tagged AtNF-YA6, AtNF-YB2 and AtNF-YB6, AtNF-YC3 and AtNF-YC7 have been produced in E. Coli and purified by Nichel-Agarose columns (Sigma). Load (L), flow-through (FT), wash (W) and eluted (E) fractions of NTA Nickel columns are shown. (TIF) [file pone.0042902.s001.tif]
